# A double shunt technique for the prevention of ischaemia of a congenital, solitary, pelvic kidney during abdominal aortic aneurysm repair: a case report

**DOI:** 10.1186/1752-1947-5-92

**Published:** 2011-03-06

**Authors:** Sotirios A Makris, Eleftherios Kanellopoulos, Anastasios Chronopoulos, Thomas G Vrachliotis, Nikolaos Doundoulakis

**Affiliations:** 1 Department of Vascular and Endovascular Surgery, Henry Dunant Hospital, 107 Mesogion Ave, Athens, 11526, Greece; 2 Department of Interventional Radiology, Henry Dunant Hospital, 107 Mesogion Ave Athens, 11526, Greece

## Abstract

**Introduction:**

Congenital solitary pelvic kidney is a rare condition, and its association with an abdominal aortic aneurysm is even more unusual. To the best of our knowledge, only two such cases have been reported in the literature to date.

**Case presentation:**

We report the case of a 59-year-old Caucasian man with a congenital solitary pelvic kidney, who was found to have an abdominal aortic aneurysm 83 mm in diameter. Abdominal computed tomography angiography clearly identified two renal arteries, one originating from the aortic bifurcation. and the other from the proximal portion of the right common iliac artery. At surgery, renal ischaemia was prevented by introduction of an axillofemoral shunt (consisting of two femoral cannulas and a vent tube of extracorporeal circulation) from the right axillary to the right femoral artery, and a second Argyle shunt from the right common iliac artery to the origin of the left renal artery. A 20 mm Dacron tube graft was then implanted. Our patient's postoperative renal function was normal.

**Conclusion:**

The renal preservation double shunt technique used in this case seems to be effective during abdominal aortic aneurysm repair.

## Introduction

Congenital solitary pelvic kidney (CSPK) has been reported to be present in 1 in 22,000 post-mortem examinations [1]. The combination of this rare renal anomaly with an abdominal aortic aneurysm (AAA) poses a therapeutic challenge because, by definition, suprarenal aortic clamping is always mandatory in these cases. Therefore, patients with these concomitant disorders are at high risk of developing renal ischaemia during conventional open repair of their aneurysm. We describe and comment on our experience of managing a case with this dual pathology.

## Case presentation

A 59-year-old Caucasian man was referred to our vascular department with a pulsatile abdominal mass around the umbilicus. He had a known history of a solitary pelvic kidney. This congenital anomaly had been diagnosed during evaluation of recurrent urinary tract infections 10 years previously.

**Figure 1:**
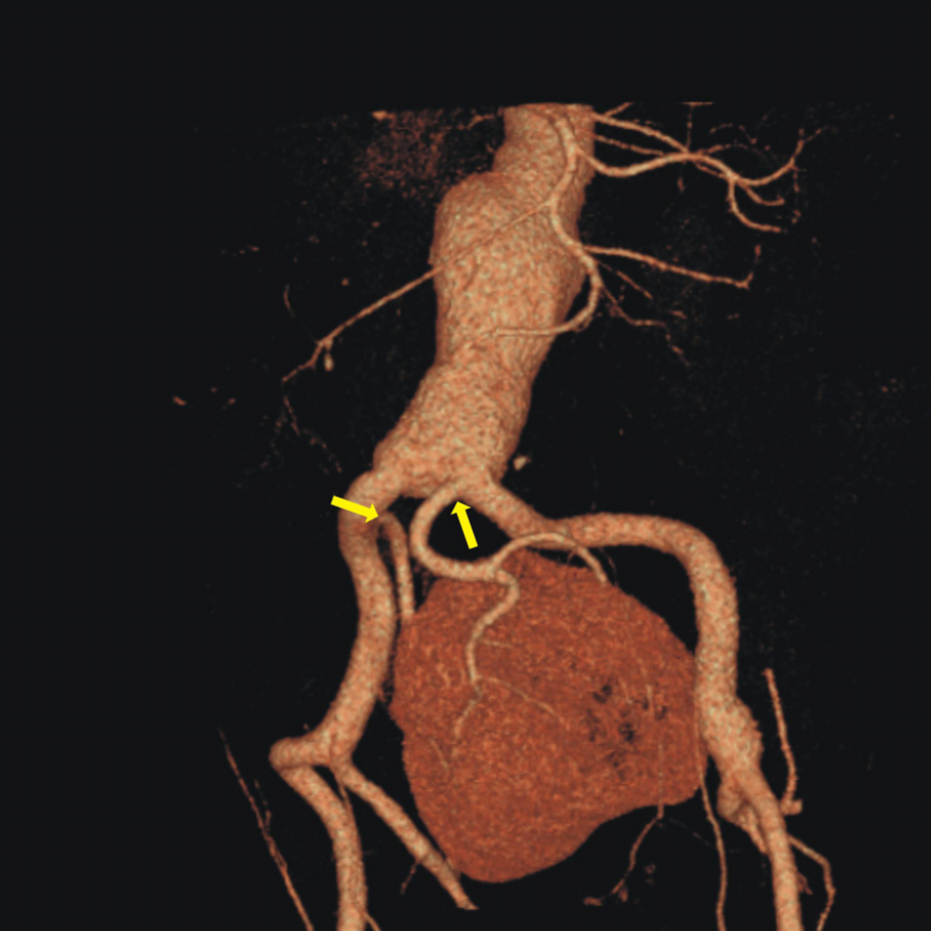
**Pre-operative computed tomography angiography of a 59-year-old patient revealed an asymptomatic abdominal aortic aneurysm, and a solitary pelvic kidney, and the composition of the renal vasculature**. One renal artery originated from the aortic bifurcation and the other from the proximal portion of the right common iliac artery (yellow arrows).

On physical examination, lower extremity pulses (that is, anterior and posterior tibial arteries) were palpable, without evidence of other arterial aneurysms. Our patient was a heavy smoker (100 pack-years; approximately 50 cigarettes/day for 40 years), and his medical history also included hypertension and hyperlipidaemia, both well controlled on monotherapy. Laboratory tests, chest radiography and electrocardiography were normal. Serum creatinine level was 1.3 mg/dL(0.16 mmol/L) [normal range 0.7 to 1.3 mg/dL (0.06 to 0.11 mmol/L)]. Computed tomography angiography (CTA) revealed an 83 mm abdominal aortic aneurysm, and verified the presence of a solitary pelvic kidney. The renal arterial anatomy was also examined; a large, predominant artery from the aortic bifurcation supplied the left portion of the renal parenchyma (hereafter referred to as 'left renal artery') and a smaller vessel (right renal artery) arose from the proximal portion of the right common iliac artery (Figure 1). An intravenous excretory pyelography was then performed, and clearly showed the single fused renal mass lying in the middle of the pelvis and containing two separate, well-formed and of normal size and course collecting systems.

**Figure 2:**
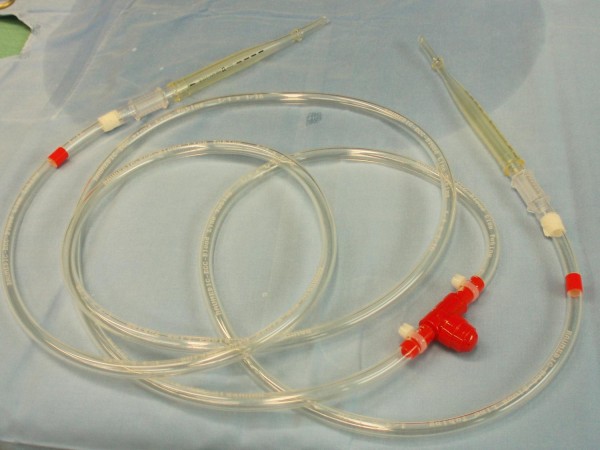
**The first shunt**. The axillofemoral shunt consisting of two 22 Fr femoral cannulas connected to each other with a half-inch Vent tube of extracorporeal circulation and an one-way valve, similar to a custom-made long Javit-like shunt used in carotid surgery.

It was apparent on the one hand that no attempt of endovascular exclusion of the aneurysm could be performed without compromising the renal blood supply. On the other hand, surgical implantation of a graft needs to be individually designed and focused on the prevention of renal ischaemia. This particular problem was solved by creating an axillofemoral shunt similar to a long Javit-like shunt used for carotid surgery. This shunt was composed of a 22 Fr femoral cannula (Edwards Lifesciences, Irvine, CA, USA), a half-inch Vent tube (Maquet Cardiopulmonary, Hirrlinger, Germany) for extracorporeal circulation with a one-way valve, followed by a second 22 Fr femoral cannula (Figure 2).

**Figure 3:**
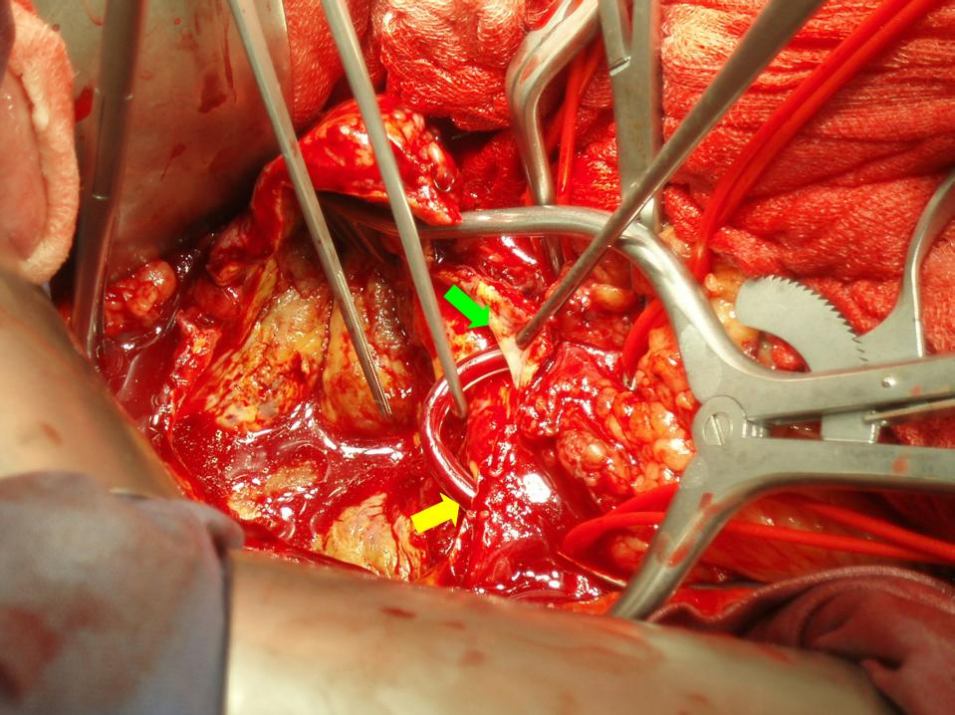
**The second shunt**. When the aneurysmal sac was opened, the left renal artery orifice was located, and an Argyle shunt was placed from the right common iliac artery (yellow arrow) to the left renal artery orifice (green arrow), allowing the whole renal parenchyma to be adequately perfused.

We performed elective resection of the aneurysm via a midline incision and transperitoneal exposure of the aorta. Both common iliac arteries and the left renal artery were dissected and controlled with vessel loops. The right axillary and common femoral arteries were then exposed via separate incisions, and the cannulae of the axillofemoral shunt were placed in them through small arteriotomies. The distal common femoral artery was clamped to improve retrograde perfusion to the kidney. Diuresis was induced with mannitol (12.5 g intravenously) and anticoagulation was initiated with intravenous heparin (7,500 U). The aorta and both common iliac arteries were cross-clamped. The right common iliac artery clamp was applied proximally to the right renal artery origin, allowing backflow perfusion of the right moiety of the renal parenchyma. The aneurysmal sac was then opened, the left renal artery orifice was identified, and a 12 Fr Argyle shunt (Tyco Healthcare, Tullamore, Co. Tipperary, Ireland) was inserted into it. The other edge of this shunt was positioned into the right common iliac artery, thus blood flow was retained to the left half of the solitary kidney (Figure 3). A 20 mm tube Dacron graft was then interposed, and the procedure was carried out without further difficulty. The selection of the graft size was based upon the wide width of the aortic bifurcation. The total time of renal ischaemia did not exceed five minutes.

**Figure 4:**
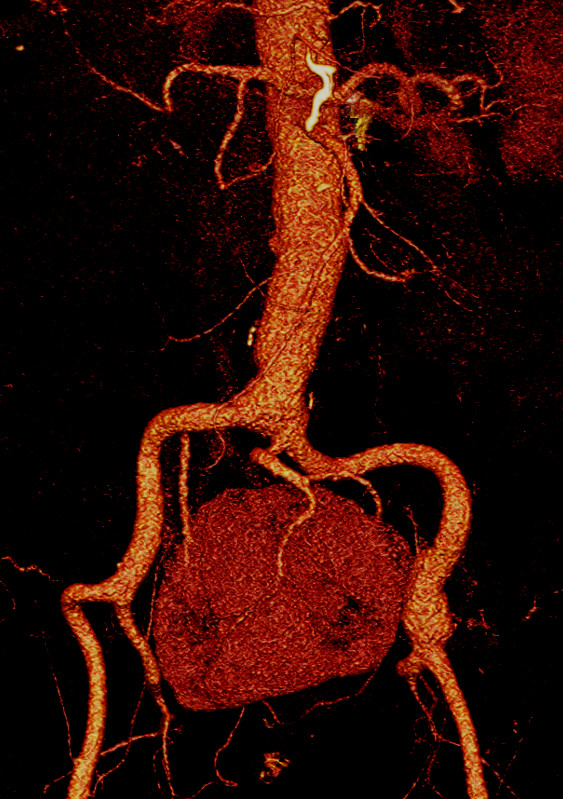
**Follow-up two years after computed tomography angiography**.

Our patient was extubated the same day in the intensive care unit. His renal function and blood pressure remained unchanged throughout his hospital stay (diuresis greater than 60 ml/hr, creatinine levels <1.1 mg/dL (0.097 mmol/L)). He had prolonged paralytic ileus, which responded well to supportive therapy, without evidence of bowel obstruction or ischaemia. Pneumonia also developed on the fourth postoperative day, but responded well to intravenous administration of antibiotics, allowing discharge on the ninth postoperative day. There was no rise in renal retention values and no deterioration of the existing hypertension during 24 months of follow-up. Subsequent CTA confirmed the successful result (Figure 4).

## Discussion

The combination of a CSPK with an AAA is exceedingly rare. Several maneuvers have been proposed to maintain blood flow to solitary, ectopic or transplanted pelvic kidneys during aortic cross-clamping. Axillofemoral [2,3] or temporary abdominal in-line (Gott) [2,4] shunts are considered an excellent solution, but their placement can be difficult and time-consuming. The double proximal clamping technique, as described by Lacombe [5] and modified by Hollis [6], represents an effective alternative, as groin incisions are avoided. However, it requires adequate patent lumbar circulation, and manipulation of the aneurysmal sac could potentially lead to distal embolization.

To the best of our knowledge, this is only the third publication in the literature describing the management of CSPK with AAA. Kaplan *et al*. first presented a patient with a single renal artery arising from the right common iliac artery [7]. The AAA was successfully repaired endoluminally with a tube stent graft, and no further attempt of preservation of renal blood flow was made. However, tube endografts are nowadays considered obsolete and have been abandoned since the early 1990 s. However, fixation of a second-generation stent graft usually necessitates the sacrifice of aberrant or accessory renal arteries originating from the aneurysmal sac or most commonly, from the iliac vessels, and therefore, minimal invasive endovascular repair cannot be used in patients with CPSK. In our case, it was obvious that the placement of the limb of a bifurcated graft or the contralateral occluder of an aortic-unifemoral device would result in disastrous renal infarction and subsequent functional impairment.

Murakami *et al *also reported a 77-year-old patient with an AAA associated with a CSPK, which was supplied by two aberrant renal arteries [8], one originating from the aortic wall just above the aortic bifurcation, and the other from the left common iliac artery. The aneurysm was repaired by interposition of a tube graft. Renal preservation during aortic cross-clamping was achieved by a combination of *in situ *cold perfusion and topical ice slush. Their patient's creatinine values did not show any significant elevation at the time of discharge. Although cooling techniques and *in situ *hypothermic perfusion should prevent renal ischaemia for more than an hour [9], we felt that shunting offered the best protection for our patient.

## Conclusion

In conclusion, the presence of CSPK in conjunction with AAA significantly complicates aortic reconstructive surgery, and demands careful planning. The optimal operative management and the appropriate method of preservation of renal blood supply must be selected individually, based on anatomic considerations and the surgeon's opinion. A variety of protective techniques, including shunting, cooling and passive use of collaterals have been used to prevent intra-operative renal ischaemia. The double shunt technique described above can be safely accomplished with favorable results.

## Competing interests

The authors declare that they have no competing interests.

## Consent

Written informed consent was obtained from the patient for publication of this case report and accompanying images. A copy of the written consent is available for review by the Editor-in-Chief of this journal.

## Authors' contributions

SAM, EK, AC and ND planned and performed the operation. TGV interpreted the CT angiography and gave specific information about the vascular anatomy of the pelvic kidney. All authors read and approved the final manuscript.
